# Dietary Probiotics Affect Gastrointestinal Microbiota, Histological Structure and Shell Mineralization in Turtles

**DOI:** 10.1371/journal.pone.0147859

**Published:** 2016-02-01

**Authors:** Mateusz Rawski, Bartosz Kierończyk, Jakub Długosz, Sylwester Świątkiewicz, Damian Józefiak

**Affiliations:** 1 Department of Animal Nutrition and Feed Management, Poznań University of Life Sciences, Poznań, Poland; 2 National Research Institute of Animal Production, Department of Animal Nutrition and Feed Science, Balice, Poland; Catalan Institute for Water Research (ICRA), SPAIN

## Abstract

Probiotics are widely used in nutrition, and their mode of action is intensively studied in mammals and birds; however, it is almost unknown in reptiles. In the present study, *Trachemys scripta scripta* and *Sternotherus odoratus* were used to assess the effects of dietary probiotics on chelonian gastrointestinal tract microecology. In the first, 20-week experiment, 40 young *T*. *s*. *scripta* were randomly distributed to four experimental groups: 1^st^, (CON)–with no additives; 2^nd^, (SSPA) with *Bacillus subtilis* PB6; 3^rd^, (MSP)–with multiple strain probiotic; and 4^th^, (SSPB) with *Bacillus subtilis* C-3102. The first study has shown that SSPA and MSP decreased the numbers of total bacteria, *Enterobacteriace*, *Staphylococcus* sp. and *Streptococcus* sp. excreted to water and increased the villous height and mucosa thickness in duodenum. SSPB improved the duodenal microstructure; however, it also increased numbers of kanamycin and vancomycin resistant bacteria, *Staphylococcus* sp. and *Streptococcus* sp., in water. In the second, 52-week experiment, 30 *S*. *odoratus* were randomly assigned to three dietary treatments. CON, SSPA and MSP groups. The MSP preparation increased the body weight gain, crude ash, Ca and P share in the turtles’ shells. Both probiotics affected duodenal histomorphology. SSPA decreased the villous height, while MSP increased the villous height and mucosa thickness, and decreased the crypt depth. SSPA decreased the concentrations of bacteria excreted to water. In the case of intestinal microbiota, bacteria suppressing effects were observed in the case of both probiotics. MSP increased the number of *Bifidobacterium* sp. and *Lactobacillus* sp.*/Enteroccoccus* sp., and decreased the number of *Clostridium perfringens* and *Campylobacter* sp. in the small intestine. In the large intestine it lowered, amongst others, *Bacteroides*–Pervotella cluster, *Clostridium leptum subgroup* and *Clostridium perfringens* numbers. The above-mentioned results suggest that probiotics are useful in turtle nutrition due to their positive effects on growth performance, shell mineralization, duodenal histomorphology and microbiota.

## Introduction

The natural history and ecology of turtles has been studied for years, and such information is available for many species. [1–3]. Currently, wild populations of this ancient group of reptiles are, on the one hand, facing the threat of extinction due to overharvesting and habitat loss. On the other hand, they are frequently farmed in the USA for the pet market, and in Asia due to their nutritional and medical use [4–7]. According to data published by Food and Agriculture Organization of the United Nations (FAO), global soft-shell turtle production reached 348 thousand tons in 2013 and is increasing rapidly, turning it into a multi-billion dollar industry [4]. However, the microbiology of the reptilian gastrointestinal tract (GIT), its composition and effects on the host still remain almost unknown and only a few studies have been published [8, 9]. Most scientific papers are concerned with reptiles only as pathogen carriers and as a zoonotic threat to people [10–13]. Salmonellosis caused by pet reptiles kept in 1–2% of households are supposed to be responsible for 6–11% of this disease in the USA [14]. In European countries, such as Germany and Poland, the reptiles are kept in similar numbers and are found in about 1% of households. [15]. The most important way of the pathogen spreading is direct contact with reptiles or their environment contaminated by feces, which is particularly hazardous for children which often play with pet turtles [15, 16]. In the present study, yellow-bellied slider turtles (*Trachemys scripta scripta*) and common musk turtles (*Sternotherus odoratus*) were used to assess the effects of probiotics on growth performance, microbiota, and the histological structure of the chelonian gastrointestinal tract. The beneficial effects of dietary probiotics have been known since ancient times [17, 18] and their nutritional properties have been widely studied in both animals and humans [18–20]. They are used in livestock nutrition to improve growth performance parameters, immune status, as well as microbiota composition [17, 21]. However, to our knowledge, there is no experimental data about their potential influence on *T*. *s*. *scripta* and *S*. *odoratus* GIT microecology and growth performance. Therefore, the presented experiments were designed to provide data, essential for understanding the mode of action of probiotics in turtle GIT. Additionally, the positive effects of probiotics on turtle GIT’s microbiota may be an important factor to improving biosecurity for their keepers and the financial result of turtle farms. The objective of this study was to evaluate the effect of dietary addition, of different, single and multiple species, of probiotic preparations on growth performance, shell composition, intestinal microbiota, and intestinal histology in *T*. *s*. *scripta* and *S*. *odoratus*.

## Materials and Methods

### Ethics statement

The study was carried out in strict accordance with the recommendations of the National Ethics Commission (Warsaw, Poland). All procedures and experiments compliedwith the guidelines and were approved by the Local Ethics Commission of the Poznań University of Life Sciences (Poznań, Poland) with respect to animal experimentation and care of animals under study, and all efforts were made to minimize suffering (Permit number: 22/2012). The turtles were obtained from a commercial pet dealer to make them a representative sample for the European pet market. The animals were euthanized by decapitation according to AVMA Guidelines for the Euthanasia of Animals [22]. Euthanasia was performed as a part of a 3-step protocol (injectable anesthetic, decapitation, pithing). The first step was an injection of ketamine (50 mg/kg IM), subsequently decapitation was performed with the use of a guillotine; to ensure death and avoid unnecessary suffering, the brain structure was destroyed by pithing.

### Animals and diets

**Fig 1:**
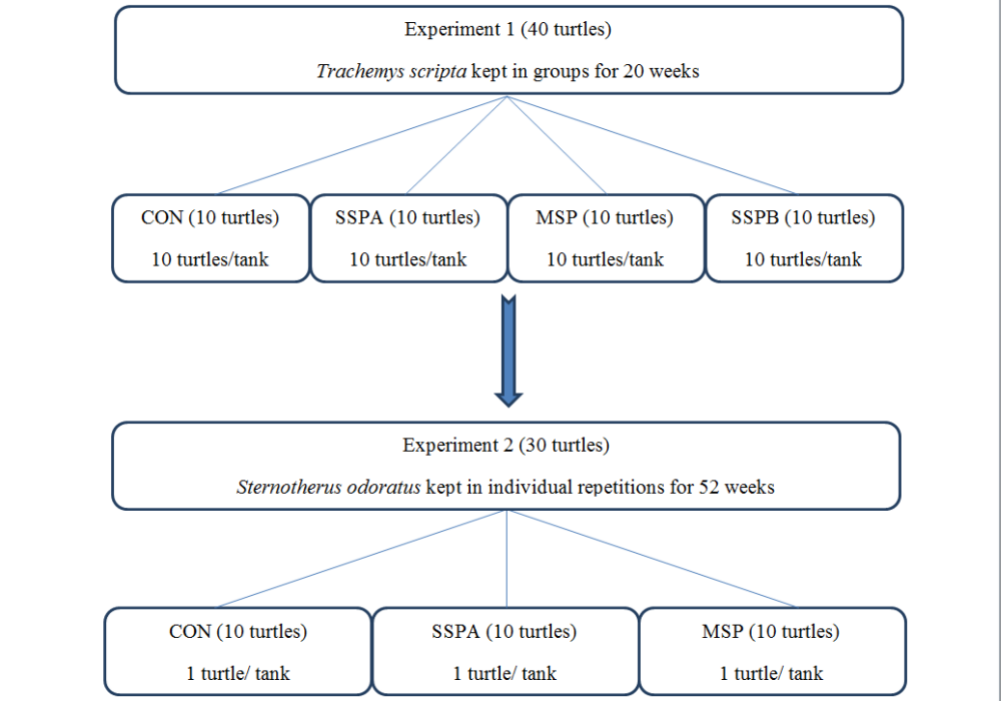
Diagram of the experimental design used in animal experiments. CON–control treatment, SSPA–single strain probiotic *Bacillus subtilis* PB6, MSP–Multiple strain probiotic, SSPB–single strain probiotic *Bacillus subtilis* C-3102.

Two long-term 20- and 52-week growth experiments were carried out on yellow-bellied slider turtles (*T*. *s*. *scripta*) and common musk turtles (*S*. *odoratus*) respectively. The design of both studies is presented in Fig 1. A preliminary 20-week study was carried out on 40 young yellow-bellied slider turtles (mean body weight 12.92 g, mean straight carapace length 40.92 mm). The animals were randomly allocated to four dietary treatments using 10 animals per treatment. Turtles were kept in plastic tanks (80x40x40 cm) filled with 80 l of water and 10 specimens were assigned to each aqua terrarium. The second 52-week experiment was carried out on 30 young common musk turtles (mean body weight 6.25 g, mean straight carapace length 31.20 mm) randomly allocated to plastic tanks (15x15x15 cm), filled with 2 l of water, treated as individual repetitions – 1 turtle per tank. The turtles were acclimatized for one month to experimental tanks and diets (with no additives). In the first week after settlement, they were fed live bloodworms (*Chironomidae*); later on in the experimental phase gelatin-based diets (Table 1). Water and air temperatures were controlled by a thermostat and maintained at a constant temperature of 28°C throughout the experiments; the water in the turtle tanks was changed every 48 h. Water used for the turtle tanks was filtered with reverse osmosis membrane filter (Aqua Art reverse osmosis system 380). During water changes the tanks were washed manually with hot water and they were scrubbed to remove algae and sediments from bottom and walls. The tanks were disinfected once weekly (without water and animals) with a UVC lamp for 30 minutes under a laminar cabinet. This method was chosen to avoid accumulation of disinfectants and its residues No chemical disinfectants were used due to their wide spectrum of adverse effects on aquatic organisms. In the authors’ opinion it is the best way for pathogen control in laboratory conditions when we are working with aquatic organisms, due to the lack of its further effects on water composition.

**Table 1:** Calculated nutritional value of the diet in dry matter.

Nutrient	Share
Crude Protein	34.55%
Crude Fat	6.91%
Crude Fiber	1.10%
Crude Ash	9.65%
Ca	4.47%
P	1.63%
Vitamin A	40000 IU/kg
Vitamin D3	8000 IU/kg
Vitamin E	80 mg/kg
Vitamin K	70 mg/kg
Biotin	200 mg/kg
Choline chloride	800 mg/kg
Fe	180 mg/kg
Mn	340 mg/kg
Zn	240 mg/kg
Cu	32 mg/kg
Fe	180 mg/kg

IU–international units

In the first experiment, the animals were assigned to 4 dietary treatments: control (CON) with no additives; single species probiotic A (SSPA)–with the addition *Bacillus subitlis* PB6: 2·10^9^ CFU/ g of preparation (Kemin Industries, USA); multiple species probiotic treatment (MSP) containing (*Lactobacillus plantarum–* 1.26·10^7^ CFU/ g, *L*. *delbruecki* subsp. *bulgaricus–* 2.06·10^7^ CFU/ g, *L*. *acidophilus–* 2.06·10^7^ CFU/ g, *L*. *rhamnosus–* 2.06·10^7^ CFU/ g, *Bifidobacterium bifidum–* 2.00·10^7^ CFU/ g, *Streptococci salivarius* subsp. *thermophilus–* 4.10·10^7^ CFU/ g, *Enterococcus faecium–* 5.90·10^7^ CFU/ g, *Aspergillus oryzae–* 5.32·10^6^ CFU/ g, *Candida pintolepessi–* 5.32·10^6^: total number of live microorganisms 2.0·10^8^ CFU/g of preparation (Probiotics International Ltd., Lopen Head, South Petherton, Somerset, UK), single species probiotic B (SSPB) containing *Bacillus subtilis* C-3102: 1·10^10^ CFU/g of preparation (Orffa International, The Netherlands). Probiotic preparations were added to the diets ‘*on top’* according to the producers’ recommendations for poultry: SSPA – 500 ppm, MSP – 500 ppm and SSPB – 100 ppm. In the second experiment, the animals were assigned to 3 dietary treatments: 1^st^—CON; 2^nd^—SSPA and 3^rd^—MSP. Probiotic preparations were used in the dosage described above.

### Measurements and sampling

The growth and development of the experimental turtles were evaluated on the basis of: body mass measurements using laboratory scale (Radwag PS 600/C/2 Radom, Poland–accuracy up to ±0.01 g) and straight carapace length (SCL) measurements using electronic calipers (accuracy up to ±0.01 mm). These measurements were used for body weight gain (BWG) and condition index (CI) calculations [23–25]. The body weight gains and condition index of turtles were measured at the end of the experiments in week 20 for *T*. *s*. *scripta* and 52 for *S*. *odoratus* respectively. The turtles were euthanized for dissection and sampling for further analyses. During dissection, GIT morphology was assessed, including lengths of: the entire tract, intestines (from duodenum to cloaca), small and large intestine. The measurements were compared to the SCL of the animal.

### Shell composition analysis

Crude Ash, Ca and P concentrations were measured in the shell (carapace and plastron). The shells were cleaned from adherent tissue and ashed (550°C for 14 h); ash weight was calculated relative to shell dry weight. The resultant ash was solubilized on a sand heater (300VC 15 min) in 10 ml 6 N HCl and 30 ml of demineralized water. The solution was transferred after filtration (ashless filters) into a 100 ml volumetric flask. The Ca and P concentrations were measured by Atomic Absorption Spectrophotometry (VARIAN Techtron AA 475, Pty. Ltd., Springvale, Australia) as described in details by Revy *et al*. (2004) [26].

### Microbiological analyses

Traditional microbiological analyses were carried out using selective agar media (Thermo Fisher Scientific Oxoid Ltd., Great Britain). Total numbers of bacteria were cultivated using Schaedler anaerobe agar (CM0437) incubated in anaerobic conditions at 35°C for 24 h. *Staphylococcus* sp. and *Streptococcus* sp. using Collumbia Agar with Sheep Blood (PB5039A) in anaerobic conditions at 36°C for 24 h. *Enterobacteriaceae* using Mcconkey agar (CM0007) incubated in aerobic condition at 35°C for 24 h. Lactic acid bacteria (LAB) using MRS (de Mann, Rugosa, Sharpe) agar (CM0316) incubated in anaerobic condition at 35°C for 48 h. Kanamycin and vancomycin resistant bacteria using Schaedler anaerobe KV selective agar (PB5204E) incubated in anaerobic condition at 35°C for 24 h. *Clostridium difficile* using Clostridium difficile agar base (CM0601). *Listeria* sp. using Brilliance Listeria Agar incubated in anaerobic condition at 37°C for 48 h. For enumeration of bacteria excreted by turtles, water samples (500 ml each) were taken after 48 h from the water change. In the case of the first experiment, 5 samples per tank were used (20 samples), in the second experiment each tank was sampled once (30 samples). Each of them was homogenized for 1 minute with 8.5 strokes/s (50 Hz) in plastic aseptic bags using stomacher homogenizer (IUL Instruments, Barcelona, Spain). One ml of sample was serially diluted in tenfold steps using a pre-reduced salt medium and cultured in 2 repetitions for each medium using 3 dilutions [27]. All plates were examined for typical colony types and for morphology characteristics associated with each growth medium.

### Microbial Community Analysis by Fluorescent *In Situ* Hybridization (FISH)

Samples of gastrointestinal content taken during turtle dissection were immediately frozen and stored in -80°C. For FISH analysis, 100 μL of digesta were diluted in PBS and pipetted onto 0.22 μm polycarbonate filters (Frisenette K02BP02500) and vacuumed (Vaccum KNF Vacuport-Neuberg). After vacuuming, the filters were transferred onto cellulose discs for dehydration in an ethanol series (50, 80, and 96%, 3 min. each). For each sample, a series of identical filters was prepared to allow the determination of optimal hybridization [28, 29]. The oligonucleotides probes used for this study (Table 2) were selected from the literature. Hybridizations were carried out in 50 μL of hybridization buffer (0.9 M NaCl; 20 mM Tris/HCl, pH 7.2; 0.01% SDS) containing the oligonucleotides probes.

**Table 2:** Oligonucleotide probes used for Fluorescent In Situ Hybridization (FISH) used for intestinal microbiota analyses in Experiment 2.

Target	Probe	Sequence (5' to 3')
*Enterobacteriaceae*	Enter1432	CTTTTGCAACCCACT^1^
*Clostridium leptum* subgroup	Clept1240	GTTTTRTCAACGGCAGTC^1^
*Streptococus* sp.*/Lactococcus* sp.	Strc493	GTTAGCCGTCCCTTTCTGG^2^
*Bifidobacterium* sp.	Bif228	GATAGGGACGCGACCCCAT^3^
*Lactobacillus* sp.*/Enterococcus* sp.	Lab158	GGTATTAGCAYCTGTTTCCA^4^
*Clostridium perfringens*	Cpref191	GTAGTAAGTTGGTTTCCTCG^5^
*Bacteroides*-Prevotella cluster	Bacto303	CCAATGTGGGGGACCTT^6^
*Akkermansia muciniphila*	Akk	ATCTGAAGCCAACCGCAAGG^7^
*Campylobacter* sp.	CAMP653	CTGCCTCTCCCTYACTCT^8^

^1^[31]

^2^[32]

^3^[33]

^4^[34]

^5^[35]

^6^[28]

^7^[36]

^8^[37]

After hybridization, filters were washed with washing buffer (20 mM Tris/HCl, pH 7.2; 0.01% SDS; 5 mM EDTA) for 20 min. at 48°C. The filters were rinsed gently in distilled water, air-dried, and mounted on object glasses with VectaShield (Vector laboratories no. H-1000) anti-fading agent containing DAPI (4',6-diamidino-2-phenylindole). To distinguish the total count (DAPI) of bacteria from other particles in ileal samples, the filters were left in 4°C for one h in the dark until visualized using a Carl Zeiss Microscope Axio Imager M2 [30].

### Histological analyses

Duodenal tissue samples were fixed immediately after dissection in freshly prepared formaldehyde solution (40 g/L of formaldehyde prepared in 0.01 *M* PBS, pH = 7.4) and incubated for 12 h. Afterwards, the samples were dehydrated in a series of alcohol solutions, placed in xylene, and then embedded in paraffin. At least 5 serial sections of 5 μm were cut from each block and were stained with haematoxylin and eosin. The stained material was examined under an Axiophot OPTON light microscope with 5×5 magnification. The length of the villi was measured from the top of the epithelium villi to the junction with the crypt. In the cross-sections, the lengths of all villi with a complete structure were measured. Destroyed villi were excluded from the experiment. The crypt depth and villous height were measured in 10 serial slides using a micrometer glass master (0.01 mm, PZO, Warsaw, Poland) and treated as the means. These values were used in further statistical analysis.

### Statistical analysis

All obtained data were tested for normal distribution using the Kolomogorow-Smirnov test. Analysis of variance was conducted using Bartlett’s test. The significance of differences among groups was determined by the Duncan’s multiple range test at the significance level of *P* ≤ 0.05. The calculations were tested using SAS 9.3 software [38]. The following general model was used: *Y_i_* = *μ* + *α_i_* + *δ_ij_*

where Y_i_ was the observed dependent variable; μ was the overall mean; α_i_ was the effect of probiotic supplementation; and δ_ij_ was the random error.

## Results

**Fig 2:**
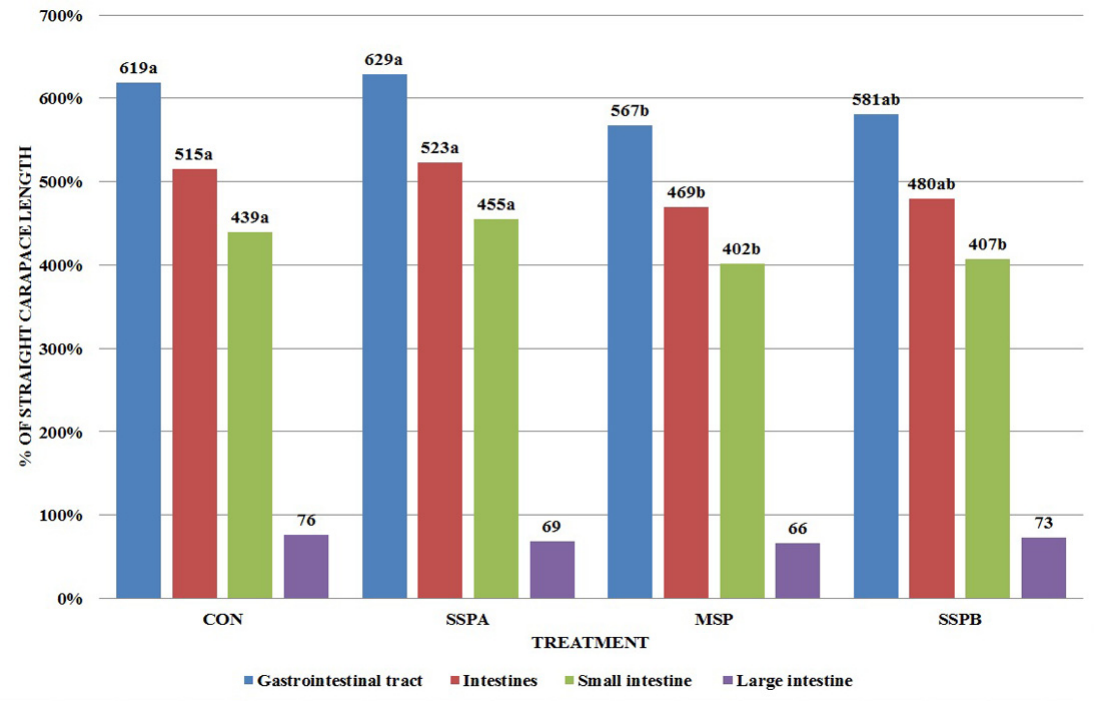
Comparison of gastrointestinal tract segments with straight carapace length (% of SCL) in *Trachemys scripta scripta* (Experiment 1). CON–control treatment, SSPA–single strain probiotic *Bacillus subtilis* PB6, MSP–Multiple strain probiotic, SSPB–single strain probiotic *Bacillus subtilis* C-3102, (*P* = 0.0427 for Gastrointestinal tract, *P* = 0.0457 for Intestines, (*P* = 0.450) for Small intestine, *P* = 0.2863 for Large intestine). Different letters indicate significant differences between treatments (P≤ 0.05).

**Fig 3:**
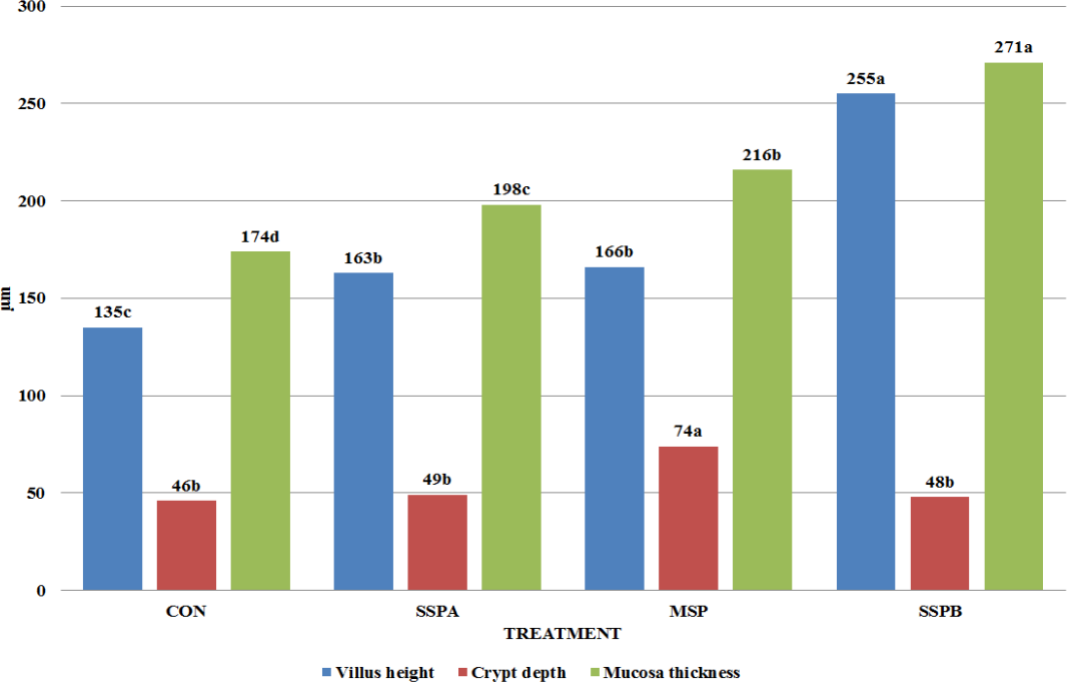
Duodenal histomorphology (μm) of *Trachemys scripta scripta* (Experiment 1). CON–control treatment, SSPA–single strain probiotic *Bacillus subtilis* PB6,. MSP–Multiple strain probiotic, SSPB–single strain probiotic *Bacillus subtilis* C-3102, (*P*<0.0001 for Villus height, Crypt depth, and Mucosa thickness). Different letters indicate significant differences between treatments (P≤ 0.05).

The results of the first experiment conducted on *T*. *s*. *scripta* are shown in Figs 2 and 3 and Table 3. None of the applied probiotics affected BWG which was: 13.6 g for CON; 14.1 g for SSPA; 13.8 g for MSP and 14.5 g for SSPB (*P* = 0.4862), and SCL gain; 9.44 mm for CON, 11.15 mm for SSPA, 10.94 mm for MSP and 10.82 mm (P = 0.9164). The condition index was: 0.51 for CON, SSPA, MSP, and 0.52 for SSPB (*P =* 0.9933).

**Table 3:** Selected microbial counts (log CFU/ml water) in water from turtle tanks determined by plate counting in the 20^th^ week of Experiment 1.

ITEM	CON	SSPA	MSP	SSPB	P
Log CFU/ ml of water					
Total number of bacteria	6.34^a^	5.72^b^	5.71^b^	6.29^a^	< .0001
Kanamycin and vancomycin resistant bacteria	4.55^b^	4.54^b^	4.43^b^	4.89^a^	0.0415
Lactic acid bacteria	5.42	5.42	5.30	5.47	0.0785
*Enterobacteriaceae*	6.36^a^	5.87^b^	5.74^b^	6.20^a^	< .0001
*Staphylococcus* sp. and *Streptococcus* sp.	5.21^b^	4.31^d^	4.89^c^	5.76^a^	< .0001

CON–control treatment, SSPA–single strain probiotic *Bacillus subtilis* PB6, MSP–Multiple strain probiotic, SSPB–single strain probiotic *Bacillus subtilis* C-3102, Different letters indicate significant differences between treatments (P≤ 0.05)

**Fig 4:**
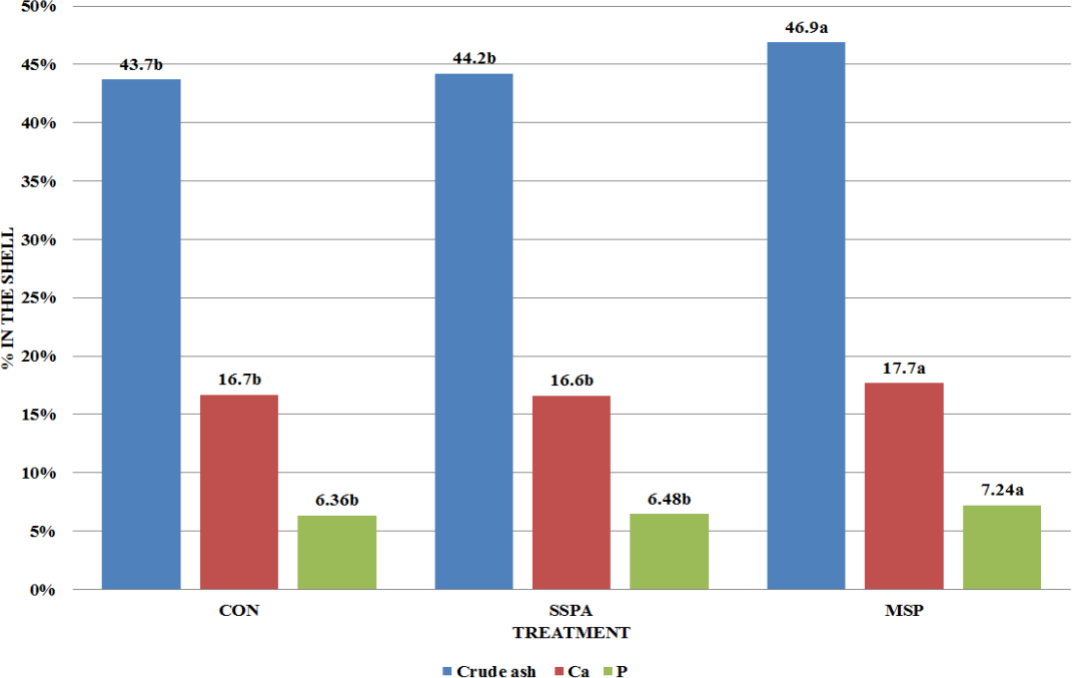
Mineral composition of the shell (%) in *Sternotherus odoratus* (Experiment 2). CON–control treatment, SSPA–single strain probiotic *Bacillus subtilis* PB6, MSP–multiple strain probiotic, (*P* = 0.0002 for Crude ash, *P* = 0.0441 for Ca, *P*<0.0001 for P). Different letters indicate significant differences between treatments (P≤ 0.05).

**Fig 5:**
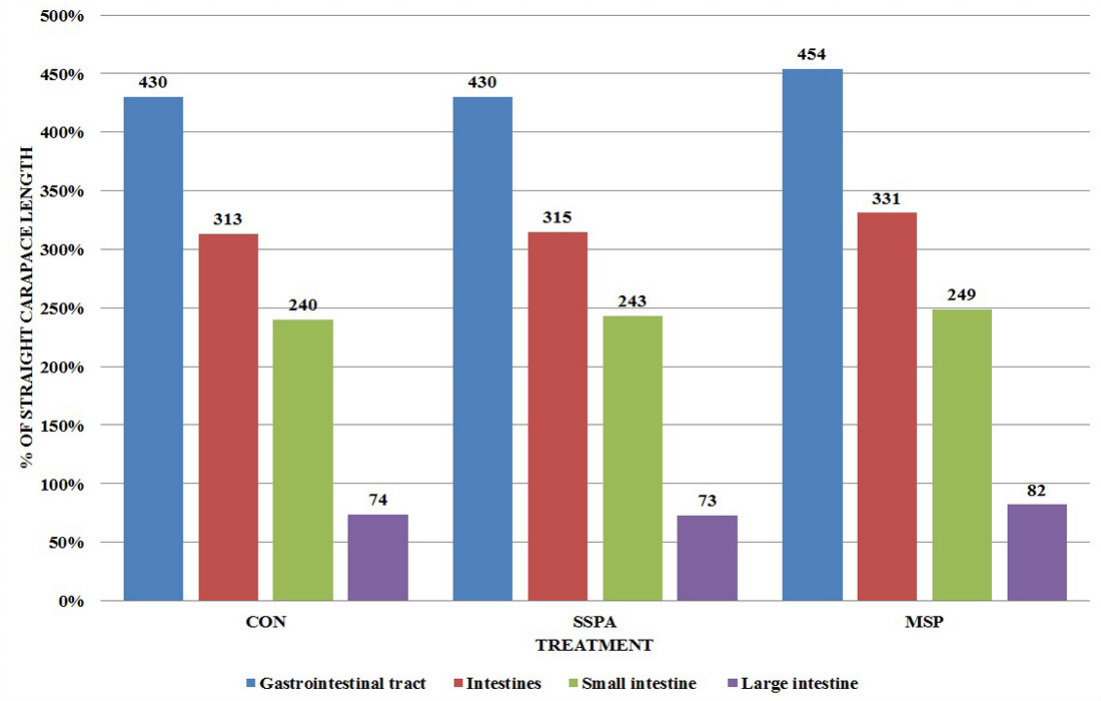
Comparison of gastrointestinal tract segments with straight carapace length (% of SCL) in *Sternotherus odoratus* (Experiment 2). CON–control treatment, SSPA–single strain probiotic *Bacillus subtilis* PB6, MSP–multiple strain probiotic, (*P* = 0.4600 for Gastrointestinal tract, *P* = 0.5678 for Intestines, *P* = 0.8765 for Small intestine, *P* = 0.5211 for Large intestine).

**Fig 6:**
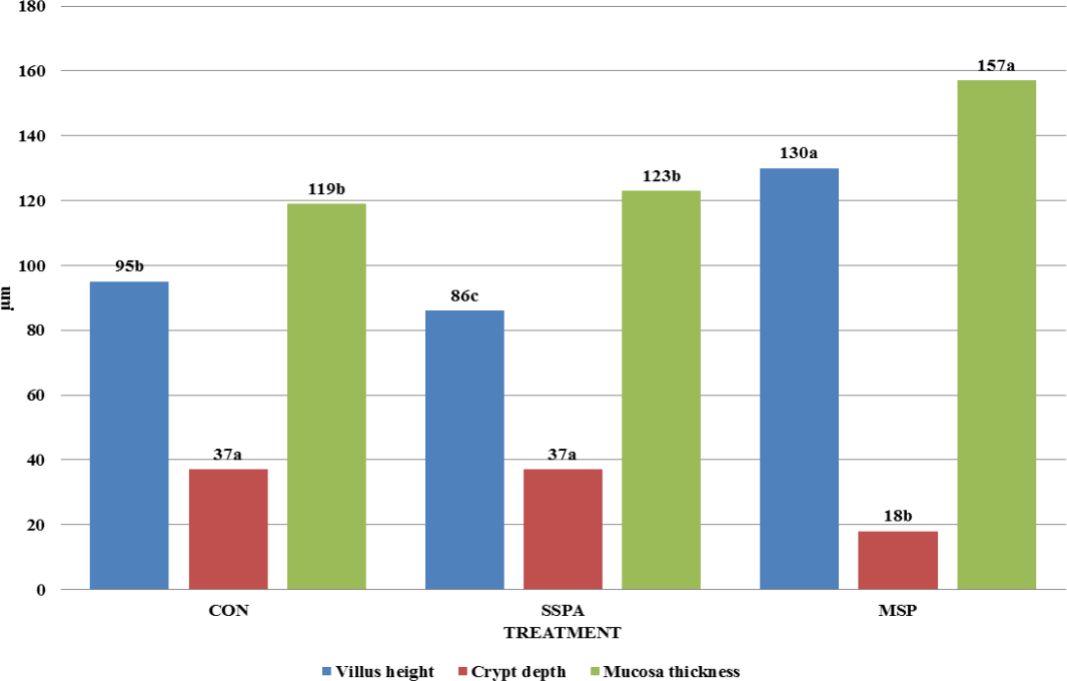
Duodenal histomorphology (μm) of *Sternotherus odoratus* (Experiment 2). CON–control treatment, SSPA–single strain probiotic *Bacillus subtilis* PB6, MSP–multiple strain probiotic, (*P*<0.0001 for Villus height, Crypt depth, and Mucosa thickness), Different letters indicate significant differences between treatments (P≤ 0.05).

The length of the entire GIT, intestines and small intestine were lowered by MSP preparation (P = 0.0427, 0.0457 and 0.0450 respectively) while SSPB use shortened length of the small intestine (Fig 2). The duodenal histological structure was affected by all dietary probiotic preparations (Fig 3). Villous height was increased in all experimental treatments, while SSPB affected it significantly higher than other probiotics (P<0.0001). Crypt depth was increased by MSP (P<0.0001). Mucosa thickness was increased by all experimental treatments (P<0.0001). In the case of microbiota excreted to water (Table 3), SSPA and MSP decreased the total number of bacteria (P<0.0001), *Enterobacteriaceae* (P<0.0001), *Staphylococcus* sp. and *Streptococcus* sp. (P<0.0001). However, in the last of the aforementioned groups, the effect of MSP was significantly lower than in SSPA treatment. *Bacillus subtilis* C-3102 (SSPB) increased the number of kanamycin and vancomycin resistant bacteria and *Staphylococcus* sp. and *Streptococcus* sp. in the water (P = 0.0415). The results of the second experiment are shown in Figs 4–6 and Tables 4–6.

**Table 4:** Selected microbial counts (log CFU/ml water) in water from turtle tanks determined by plate counting (Experiment 2).

ITEM	CON	SSPA	MSP	*P-value*
log CFU/ml of water				
Total number of bacteria	5.13^ab^	4.77^b^	5.50^a^	0.0324
Kanamycin and vancomycin resistant bacteria	5.39^a^	4.70^b^	5.69^a^	0.0003
Lactic acid bacteria	4.26	3.95	4.31	0.3068
*Enterobacteriaceae*	5.45^a^	4.59^b^	5.15^ab^	0.0033
*Staphylococcus* sp. and *Streptococcus* sp.	4.45^a^	3.70^b^	4.28^a^	< .0001
*Clostridium difficile*	4.50^a^	3.42^b^	4.15^ab^	0.0013
*Listeria* sp.	5.91	5.48	5.48	0.3733

CON–control treatment, SSPA–single strain probiotic *Bacillus subtilis* PB6, MSP–multiple strain probiotic, Different letters indicate significant differences between treatments (P≤ 0.05)

**Table 5:** Selected microbial counts (log CFU/g digesta) in small intestine digesta determined by DAPI staining and fluorescent in situ hybridization (Experiment 2).

ITEM	CON	SSPA	MSP	*P-value*
	log CFU/ g of digesta			
Total number of bacteria	9.90^a^	9.44^b^	9.44^b^	< .0001
*Bacteroides*-Prevotella cluster	9.03^a^	8.22^b^	8.38^b^	< .0001
*Clostridium leptum* subgroup	8.82^a^	8.51^b^	8.41^b^	0.0002
*Enterobacteriaceae*	8.60^a^	8.28^b^	8.04^c^	< .0001
*Streptococcus sp*.*/Staphylococcus sp*.	8.71	8.47	8.53	0.0520
*Clostridium perfringens*	7.10^a^	7.30^a^	6.64^b^	< .0001
*Bifidobacterium* sp.	7.45^c^	7.67^b^	8.03^a^	< .0001
* Lactobacillus* sp.*/Enterococcus* sp.	7.67^b^	7.45^b^	8.05^a^	< .0001
*Akkermansia muciniphila*	7.22^a^	7.20^a^	6.87^b^	0.0114
*Campylobacter* sp.	7.59^a^	7.56^a^	6.72^b^	< .0001

CON–control treatment, SSPA–single strain probiotic *Bacillus subtilis* PB6, MSP–multiple strain probiotic, Different letters indicate significant differences between treatments (P≤ 0.05)

**Table 6:** Selected microbial counts (log CFU/g of digesta) in large intestine digesta determined by DAPI staining and fluorescent in situ hybridization (Experiment 2).

ITEM	CON	SSPA	MSP	*P-value*
	log CFU/ g of digesta			
Total number of bacteria	10.34	10.26	10.40	0.2208
*Bacteroides*-Prevotella cluster	8.86^a^	8.97^a^	8.64^b^	0.0005
*Clostridium leptum* subgroup	8.98^a^	9.03^a^	8.67^b^	< .0001
*Enterobacteriaceae*	9.13^a^	8.84^b^	8.75^b^	< .0001
*Streptococcus sp*.*/Staphylococcus sp*.	8.97^a^	8.72^b^	8.76^b^	0.0033
*Clostridium perfringens*	9.03^a^	8.94^a^	8.75^b^	0.0013
*Bifidobacterium* sp.	9.19^a^	8.90^b^	8.95^b^	0.0004
* Lactobacillus* sp.*/Enterococcus* sp.	8.70	8.65	8.73	0.6019
*Akkermansia muciniphila*	7.96^a^	7.63^b^	7.62^b^	< .0001
*Campylobacter* sp.	9.06^a^	8.72^b^	8.78^b^	0.0003

CON–control treatment, SSPA–single strain probiotic *Bacillus subtilis* PB6, MSP–multiple strain probiotic, Different letters indicate significant differences between treatments (P≤ 0.05)

Turtle BWG in the treatment with MSP (7.22 g) was significantly higher (P = 0.0449) in comparison with the CON (5.63 g) and SSPA group (5.96 g), SCL gain was 5.26 mm for CON, 6.24 mm for SSPA and 6.82 mm for MSP (P = 0.4537). None of the used probiotics affected the condition index (0.30 for CON and SSPA; 0.33 for MSP; P = 0.5925). In the case of shell mineral composition MSP increased crude ash, Ca and P content (P = 0.0002, 0.0441 and <0.0001 respectively) (Fig 4). Gastrointestinal tract morphology was not affected (Fig 5). However, significant effects were observed in the case of duodenal histomorphology (Fig 6). Dietary *Bacillus subtilis* PB6 (SSPA) decreased villous height in comparison, to CON and MSP treatments (P<0.0001). The MSP preparation increased villous height, and mucosa thickness, while it decreased crypt depth in comparison with the control group (P<0.0001). In the case of bacteria excreted to water SSPA supplementation caused a significant decrease in most of the studied bacterial groups including: total number of bacteria (P = 0.0324), *Enterobacteriaceae* (P = 0.0033), kanamycin and vancomycin resistant bacteria (P = 0.0003), *Clostridium difficile* (P = 0.0013), *Staphylococcus* sp. and *Streptococcus* sp. (*P*<0.0001), (Table 4). Concentrations of microbiota in the small intestine analyzed by FISH were decreased by both probiotics in the cases of total number of bacteria (P<0.0001), *Bacteroides*–pervotella cluster (P<0.0001), *Clostridium leptum* subgroup (P = 0.0002), and *Enterobacteriaceae*, in whose case the MSP probiotic exhibited a significantly higher effect than *B*. *subtilis* PB6 (P < .0001) (Table 5). Lower bacteria numbers were also recorded in MSP treatment in terms of *Clostridium perfringens* (P<0.0001), *Akkermansia municiphila* (P = 0.0114), and *Campylobacter* sp. (P<0.0001). Both probiotics increased the number of *Bifidobacterium* sp. (P<0.0001). However, a significantly higher increase was recorded in MSP than in SSPA. Moreover, the number of *Lactobacillus* sp. and *Enterococcus* sp. was increased by MSP. In the case of large intestine microbiota populations (Table 6) we observed no difference among treatments in total bacterial count, *Lactobacillus* sp. and *Enterococcus* sp. Both probiotics decreased concentrations of *Enterobacteriaceae* (P<0.0001), *Streptococcus* sp.*/Lactococcus* sp. (P = 0.0033), *Bifidobacterium* sp. (P = 0.0004), *Akkermansia municiphila* (P < .0001) and *Campylobacter* sp. (P = 0.0003), while MSP additionally lowered the numbers of *Bacteroides–*Prevotella cluster (P = 0.0005), *Clostridium leptum* subgroup (P<0.0001) and *Clostridium perfringens* (P = 0.0013).

## Discussion

In the available literature there are several modes of dietary probiotic actions assumed to take place in animal GIT: competition with pathogenic bacteria for intestinal adhesion sites and nutrients; enhancement for epithelial barrier integrity; production of antimicrobial substances including bacteriocins; microbial enzymes; modifications of environmental conditions in the intestine by lowering pH through the increased production of organic acids and enhancement of the intestinal immune function [17, 18, 21, 39, 40]. In both experiments it was observed that dietary probiotics affect turtle GIT microbiota. However, only in the second experiment was turtle BWG improved by MSP preparation.The lack of effects of other preparations on growth performance is probably related to the high specificity of mutual relations between strains used as probiotics and animal host species. It may be assumed that the ability of attachment to epithelial cells in the GIT is strain and host specific [17]. In the first experiment, SSPA and MSP treatments had bacteria suppressing effects. However, in comparison with the above SSPB treatment increased the numbers of potentially pathogenic *Enterobacteriaeae*, *Staphylococcus* sp. and *Streptococcus* sp. and kanamycin and vancomycin resistant bacteria. All probiotic preparations significantly improved villous height and mucosa thickness in the duodenum of *T*. *s*. *scripta*. However, in the case of SSPB, the effect was also significantly higher than in SSPA and MSP preparations. It suggests that GIT microstructure development might be linked to fecal microbiota numbers for which it constitutes an environmental niche. The relation of the GIT epithelium development to the number of excreted microbiota was also observed in the second experiment where reduced total bacteria excreted to water were observed in SSPA treatment, which was characterized by decreased villous height. Higher levels of excreted *Enterobacteriaceae* in SSPB treatment (the first experiment) may lead to a zoonothical threat for turtle keepers and increased pathogen pressure for animals [41]. Additionally, an increased level of kanamycin and vancomycin resistant bacteria may lead to antibiotic resistance spreading in the environment [18]. Therefore, although SSPB in poultry and pigs is considered as an effective probiotic, in the case of turtles it may not meet all the required criteria for proper probiotic *in vivo* action [42, 43]. In the second experiment, microbiota modification, histomorphological structure alteration, growth promotion and shell composition improvement, as the effect of diet supplementation with probiotic preparations, were observed. The MSP seems to be the most effective in the case of *S*. *odoratus*, while it affects beneficially all of the above mentioned aspects. Improvement in BWG and shell mineralization suggests improved nutrient utilization due to MSP supplementation as compared to the CON and SSPA treatments. The quantitative reduction of microbiota may be one of the main reasons for higher BWG, while decreased bacteria concentration in the GIT is well described in the literature as a growth promoting factor, mainly due to reduced competition between the host and microbiota for nutrients [30, 44, 45]. Moreover, lower counts of potentially pathogenic bacteria together with the increase of probiotic bacteria concentrations are key factors for microbial balance, intestinal health and gut integrity when probiotics are used [39]. In the case of the small intestine, MSP treatment lowered counts of total bacteria, *Clostridium perfringens*, *Streptococcus* sp. and *Staphylococcus* sp., *Enterobacteriaceae*, and *Campylobacter* sp. The decrease of these potentially pathogenic bacteria was simultaneous with *Bifidobacterium* sp. and *Lactobacillus* sp./*Enterococcus* sp. population increase. It suggests that strains of *Bifidobacterium bifidum*, *Lactobacilli and Enterococci* from the MSP preparation inhabited turtle GIT or promoted their native probiotic populations. *Bifidobacteria* in the GIT may produce acetic and lactic acids at a ratio of 3:2 which are effective against gram-negative pathogens like *Enterobacteriaceae* or *Campylobacter* sp. [18]. *Clostridia* and other gram-positive bacteria were probably suppressed by direct microbial competition for adhesion sites with probiotic strains (competitive exclusion) and bacteriocin production, which seem to be important mechanisms of lactic acid bacteria action [18, 40, 46]. An interesting finding is probably the first case of *Akkermansia muciniphila* detection in the turtle gastrointestinal tract. It is a commensal representative of *Verrucomicrobiaceae* involved in mucin degradation which seems to be a common inhabitant in humans and animals, including reptiles (*Python morulus*) [47–49]. However, it was probably overlooked in previous studies on the microbiota of many species, due to its inconspicuous cell morphology, small size and specific carbon source requirements [36, 47]. In the presented experiment on *S*. *odoratus*, the levels of *A*. *muciniphila* were lowered by SSPA in large intestine and by MSP in the small and large intestine. The above-mentioned results are in agreement with a study on mice in which it was revealed that probiotic treatment ameliorates metabolic syndrome symptoms, as well as increases *Lactobacillus* sp. and *Bifidobacterium* sp. and decreases *Clostridiaceae*, *Akkermansia* sp. and *Escherichia coli* [50]. It is suggested that *A*. *muciniphila* play an important role in obesity prevention, intestinal integrity development and regulation of mucus layers by utiliziation of mucin as the energy source and stimulation of growth of symbionts [51, 52]. However, it was also found that in gnotobiotic mice, the administration of *A*. *municiphila* exacerbates gut inflammation and supports the development of *Salmonella* Typhimurium infection [53]. In the authors’ opinion, the role of this interesting microbe in the animal microbiota is strongly connected with the species of the host animal and the condition of its microbiota. Further studies should be undertaken to reveal the circumstances in which *A*. *municiphila* is a symbiotic or potentially harmful bacteria and what its mode of action is in the microbiota and immune development. Another important mode of probiotic action which may explain BWG promotion by MSP treatment is intestinal epithelium structure and integrity enhancement [54]. Villous height and mucosa thickness increased in the above mentioned treatment. This is consistent with the fact that villous height is widely considered as a good indicator of intestinal function as their height reflects digestive capacity and absorption surface of the GIT, which may result in better growth performance [54, 55]. The presented results agree with a study on Chinese softshell turtles (*Pelodiscus sinensis*), which has shown that the probiotic strain of *Bacillus subtilis* increased BWG, improved feed conversion ratio (FCR), and caused higher activities of digestive enzymes [56]. Positive modulation of GIT microbiota was also recorded in the mentioned experiment: *Bifidobacteria* increased and *Enterobacteriaceae* decreased in the ileal digesta [56]. Similarly, the most recent study carried out on Chinese softshell turtles demonstrated the positive effects of *Bacillus subtilis* on turtle growth performance, including BWG, FCR and daily growth, as well as enzymatic activity of sucrase, maltase, amylase, lipase and ATPase in the GIT [56]. Pyrosequencing of intestinal microbiome showed lower microbial diversity and an increase of Firmicutes due to antimicrobial compounds production by *B*. *subtilis* [57]. It is consistent with our theory which suggests that, in turtles, microbiota quantitative and qualitative reduction may lower direct competition within the host and bacteria for nutrients and result in better growth performance. Irrespective of the discussed data, in our opinion, the most important result for practical turtle management, seems to be the fact that MSP had a positive effect on shell mineralization. The increased share of crude ash followed by Ca and P concentrations in the shell composition may be crucial for turtle health. Most of nutritional and metabolic issuesin captive care and breeding of chelonians are connected to shell development disturbances caused by mineral compounds malabsorption or deficiency in the diet.It may lead to Nutritional Metabolic Bone Disease (NMBD), poor growth and carapace deformations. It occurs frequently in young fast-growing reptiles in the first years of their life [58, 59]. In the second experiment, we showed not only better growth or shell mineralization, but an improvement in both parameters. It may suggest that the long-standing assumption that Ca absorption in reptiles is mainly dependent on diet composition may be incomplete [58]. It could be suggested that the increased mineral concentrations in the shell may be caused by histological microstructure alteration, i.e. increased absorption area, and suppression of pathogens in the GIT. In our opinion, with the exception to the above mentioned factors: short chain fatty acids (SCFA), vitamins and/or bacterial phytase production by probiotic bacteria might increase the bioavailability of mineral compounds during the second experiment [60]. It was earlier suggested in the literature as an additional attribute of dietary probiotics and prebiotics [60, 61]. The promotion of well-balanced growth of turtles without nutritional metabolic diseases is one of the most important aspects of their captive management. Due to the above mentioned results, dietary probiotics seem to be effective tools for microbial community stabilization in reptiles and their sustainable development promotion. However, the selection of suitable preparations appears to be crucial. All probiotic preparations used in the experiments were previously proven as effective tools in farm animals [42, 62, 63]. In the case of turtles, for all of them we have observed their effects at levels of microbiota and duodenal microstructure. However, only MSP in *S*. *odoratus* affected growth performance, which suggests that probiotic preparation effectiveness is strain and species-dependent. In our opinion, in further studies, specific strains isolated from turtles should be verified as potential probiotics to increase the probability of their colonization success in turtle GIT. If bacteria isolated from other animal species are used, multiple species preparations seem to provide a wider opportunity for finding strains beneficial for reptiles. They may be more effective and beneficial due to synergistic effects of different strains of probiotic bacteria, each with its own traits and properties for the inhibition of pathogens, and a higher chance for positive alteration of GIT function [64]. For many turtle species, large-scale farming and captive breeding programs are developed. In these cases, proper microbiota development in young animals and lower pathogen frequency should be considered as an important element of health prevention. Additionally may play an important role in biosecurity when the zoonotic threat for keepers is considered. Increased meat yield form turtle farms may decrease overharvesting of natural populations in Asia. Additionally, better growth performance and shell mineralization in turtles reared for releasing them to the wild may increase their survival rate in the natural environment. The above-mentioned reasons are crucial for further studies on turtle microbiota which should be undertaken in the future.
